# Difference in excess mortality during the COVID-19 pandemic depending on marital status in Japan

**DOI:** 10.1371/journal.pone.0354263

**Published:** 2026-07-17

**Authors:** Tasuku Okui

**Affiliations:** Medical Information Center, Kyushu University Hospital, Fukuoka, Japan; King Abdulaziz University Faculty of Medicine, SAUDI ARABIA

## Abstract

Differences in excess mortality during the coronavirus disease 2019 pandemic across marital statuses in Japan were investigated using data from across the country. Mortality data from the Vital Statistics records spanning 2010–2023 were utilized. Age-standardized mortality rates were computed by sex, marital status, year, and cause of death, and expected post-pandemic rates were estimated using pre-pandemic values with a quasi-Poisson regression model. Furthermore, the age-standardized percentage of the excess number of deaths relative to the expected number of deaths (hereafter, P-score) after the pandemic started was calculated by sex, marital status, and cause of death, using the expected and observed number of deaths. A declining trend in all-cause mortality rate was observed across all groups before the pandemic, regardless of sex and marital status, while mortality rates increased across all groups after the pandemic began. The largest decrease in all-cause mortality rate was observed among never-married persons before the pandemic in both men and women, while the smallest increase after the pandemic was observed among married persons. The highest age-standardized P-score for all-cause mortality was observed among never-married men and women, while that for married persons was the lowest among men. For women, the age-standardizedP-scores were closer across groups, with confidence intervals overlapping among marital-status categories. In contrast, there was a significant difference in the age-standardized P-scores between never-married and married persons among women aged <65 years. In conclusion, the percentage of excess all-cause mortality was the highest among never-married persons during the pandemic period in Japan, particularly in men, and it is important to continue to monitor the trend in the future.

## Introduction

The coronavirus disease 2019 (COVID-19) pandemic has affected all aspects of daily life, including social activities and health worldwide [1–3]. In the medical sector, excess mortality occurred during the pandemic in Japan and other countries [4–6], and some studies showed that the degree of excess mortality differed by sociodemographic characteristics, including region and educational attainment [7–9]. In addition, it was suggested that persons with lower socioeconomic status were more vulnerable to the pandemic’s effects [9,10].

Conversely, few studies have explored excess mortality related to the pandemic based on marital status. Marital status continues to be a significant risk factor for mortality in Japan and other countries [11,12], as persons’ health status and behaviors vary considerably depending on their marital situation [13–15]. Furthermore, the pandemic’s effects differed by marital status, and lifestyle, physical, and mental health changes during this period varied by living arrangements or marital status [16–18]. Therefore, it is valuable to investigate how the pandemic’s impact on excess mortality may have differed across marital statuses. A Korean study found that the highest excess all-cause mortality occurred among never-married persons [9]. Similarly, a prior study from Japan covering 2020–2022 reported that the mortality risk was greatest for never-married persons [19]. In contrast, even before the pandemic, the mortality rate for never-married persons was higher than that for married persons in Japan [20]. Therefore, whether the extent of excess mortality caused by the pandemic varies with marital status has not yet been studied in Japan. Additionally, the cause-specific mortality rates for diseases like cardiovascular and respiratory illnesses by marital status have not been examined since the start of the pandemic. This study examined how excess mortality during the COVID-19 pandemic varied by marital status in Japan.

## Materials and methods

We utilized Japan’s entire mortality data from the Vital Statistics, provided by the Ministry of Health, Labour and Welfare under Article 34 of the Statistics Act. The data were provided on February 5^th^ 2026, and the author did not have access to information that could identify individual participants during or after data collection. Specifically, data on persons aged 15 and older, categorized by age group, sex, month, year, cause of death, and marital status, were used, covering mortality from 2010 to 2023. In addition to all-cause mortality, we analyzed cause-specific deaths, including malignant neoplasms, cardiovascular diseases, respiratory diseases, and ill-defined causes (symptoms, signs, and abnormal clinical and laboratory findings not elsewhere classified), given the relatively high number of deaths in these categories. The International Classification of Diseases (ICD-10) codes for these causes are C00–C97, I00-I99, J00–J99, and R00–R99, respectively. Marital status categories include married, never married, widowed, and divorced. Population data segmented by sex, age group, marital status, and year for 2010, 2015, and 2020 were sourced from the Census [21]. For years without a Census, populations were estimated using linear interpolation by sex, age group, and marital status; from 2021 to 2023, population estimates were derived by linear extrapolation using data from 2015 and 2020. Since persons aged 85 or older were grouped in the population data, their mortality data were similarly aggregated. Additionally, because cause-specific death counts for some marital statuses were small among those under age 50, persons aged 15–49 were combined into a single age group. In addition, April 2020 was defined as the time point at which the pandemic began in this study, as the first state of emergency declaration was issued in Japan at that time [22,23].

The annual percent change (APC) in mortality rates before and after the pandemic was calculated separately by sex, marital status, and cause of death. Specifically, a quasi-Poisson regression model was employed with the number of deaths as the outcome variable. The model included month, age group, and a variable representing the number of months from the start of each period as explanatory variables. The logarithm of the population was used as an offset term. Along with APC, the 95% confidence interval (CI) and p-value were also computed; a p-value of less than 0.05 was considered statistically significant.

Additionally, age-standardized mortality rates were calculated by sex, marital status, year, and cause of death, using the total population of 2020 as the standard. Expected age-standardized mortality rates after the pandemic started were estimated using pre-pandemic data. In this analysis, a quasi-Poisson regression model was used, with month and time point (the number of months from the start) as explanatory variables. The model was applied across different subgroups based on age group, sex, marital status, and cause of death. To address autocorrelation in the observations, the Newey-West variance was employed in all the regression analyses in this study [24,25]. The expected age-standardized mortality rate was calculated from the expected values for each age group. The 95% CI was derived using a simulation based on a multivariate normal distribution, with the coefficients and their variance–covariance matrix serving as the mean vector and the variance matrix, respectively. In addition, the excess mortality rate per 100,000 person-years after the onset of the pandemic and its age-standardized value were calculated using the observed and expected deaths.

Additionally, we calculated the percentage of the excess number of deaths relative to the expected number of deaths (hereafter, P-score) after the pandemic by sex, marital status, and cause of death [26–28], and it was calculated by dividing the difference between the observed and expected number of deaths by the expected deaths. The P-score was shown in a percentage, and the P-score of 10% indicates that the observed number of deaths was 10% higher than the expected deaths, for example. The expected deaths in the post-pandemic period were obtained by summing the expected counts across all age groups. The 95% CI for the P-score was also computed using a simulation based on the multivariate normal distribution. In addition, the age-standardized P-score was calculated based on an average of the age group-specific P-score weighted by the age group-specific expected deaths of all the population after the onset of the pandemic [26]. Moreover, age-specific analysis was conducted for all-cause mortality, and the P-score and excess mortality rate were calculated by age group (<65 years and 65 years or older). Furthermore, the excess mortality was calculated using January 2020 as the time point at which the pandemic began as a sensitivity analysis. All statistical analyses were conducted in R4.5.0 [29], using the packages ggplot2, ggpubr, lmtest, MASS, and sandwich. During the preparation of this work, the author used Google AI Mode to check English grammar. After using this tool, the author reviewed and edited the content as needed.

This study was conducted in accordance with the Declaration of Helsinki. An approval by an institutional ethical committee was not needed because only aggregate data of the official statistics were used.

## Results

**Fig 1:**
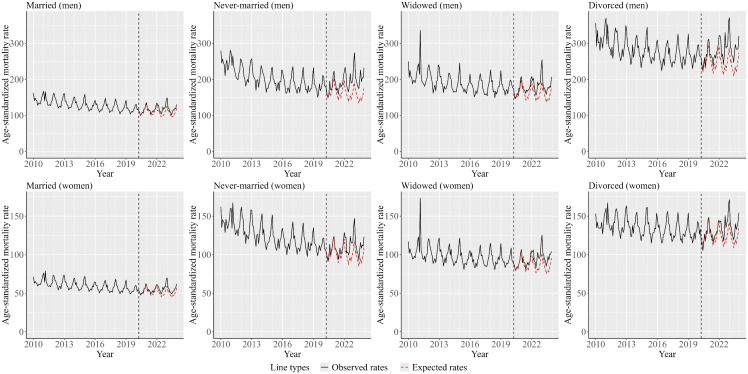
Monthly age-standardized all-cause mortality rates along with the expected values following the start of the pandemic by sex and marital status. The solid line shows the observed rate, while the vertical dotted line marks the start of the pandemic. The red dashed line represents the expected rates based on pre-pandemic data, with the shaded areas indicating the 95% CI.

Fig 1 depicts the monthly age-standardized all-cause mortality rates and their expected values following the start of the pandemic, broken down by sex and marital status. The age-standardized rate for never-married persons declined before the pandemic but showed an increasing trend afterward, and an increasing trend was observed also in widowed and divorced persons. After the pandemic began, the observed rates tended to exceed the expected rates, regardless of marital status.

**Fig 2:**
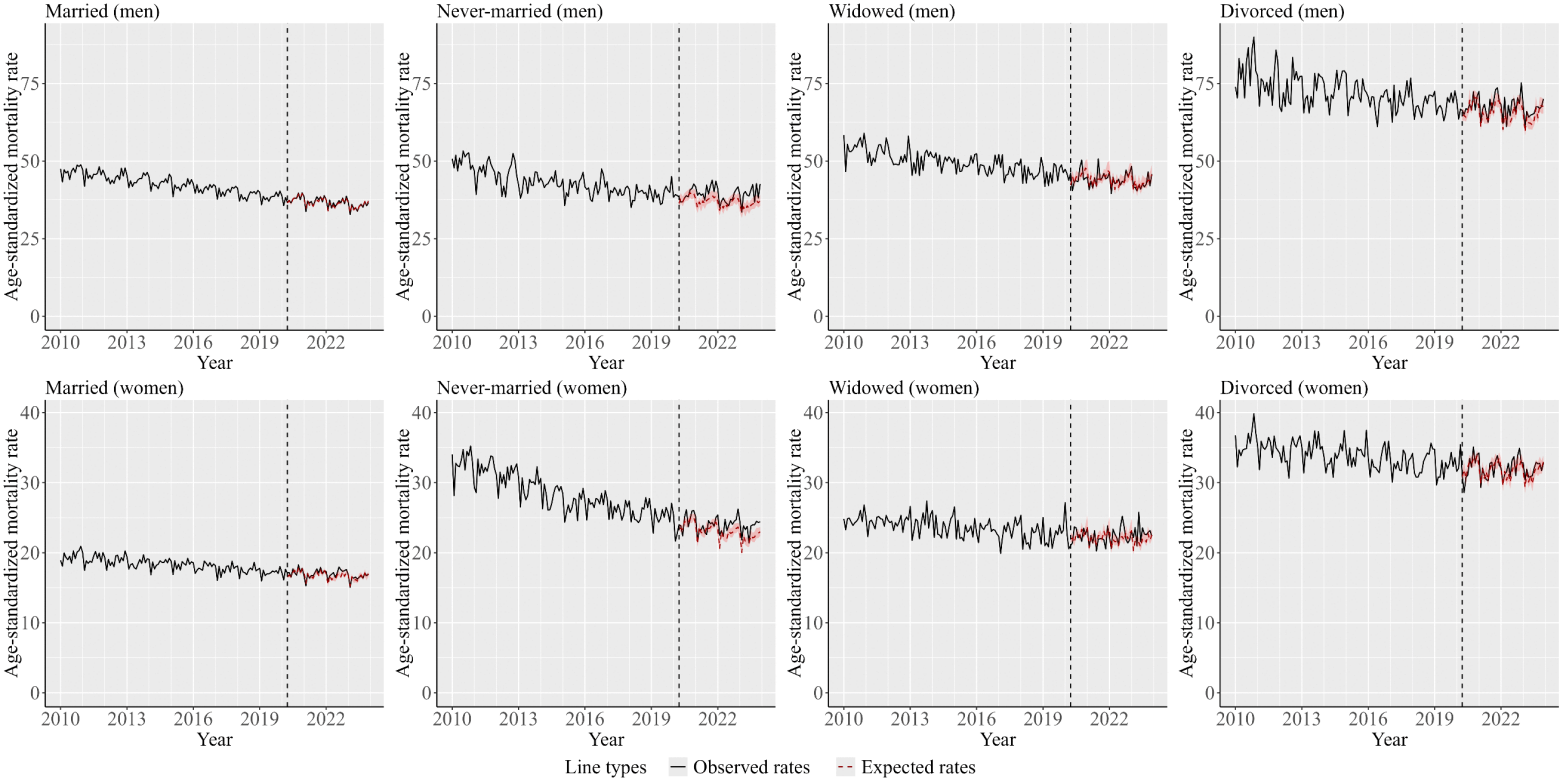
Monthly age-standardized mortality rates of malignant neoplasms along with the expected values following the start of the pandemic by sex and marital status. The solid line shows the observed rate, while the vertical dotted line marks the start of the pandemic. The red dashed line represents the expected rates based on pre-pandemic data, with the shaded areas indicating the 95% CI.

Fig 2 displays the monthly age-standardized mortality rates of malignant neoplasms along with their expected values following the outbreak of the pandemic, categorized by sex and marital status. Overall, the gap between observed and expected rates remained minimal regardless of marital statuses and sex.

**Fig 3:**
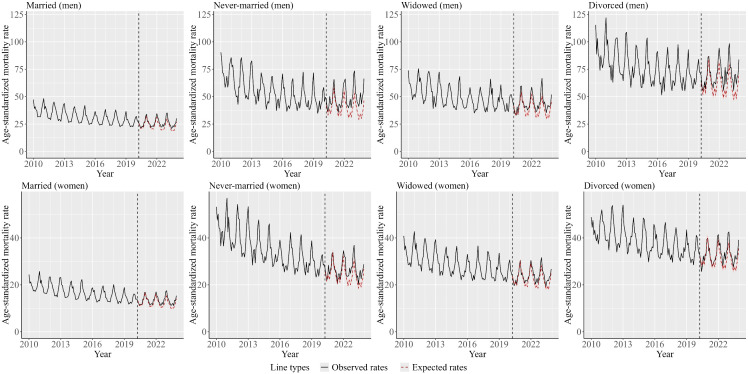
Monthly age-standardized mortality rates of cardiovascular diseases along with the expected values following the start of the pandemic by sex and marital status. The solid line shows the observed rate, while the vertical dotted line marks the start of the pandemic. The red dashed line represents the expected rates based on pre-pandemic data, with the shaded areas indicating the 95% CI.

Fig 3 illustrates the monthly age-standardized mortality rates for cardiovascular diseases and their expected values since the pandemic started, categorized by sex and marital status. The observed rates generally exceeded the expected rates during winter among never-married, widowed, and divorced persons.

**Fig 4:**
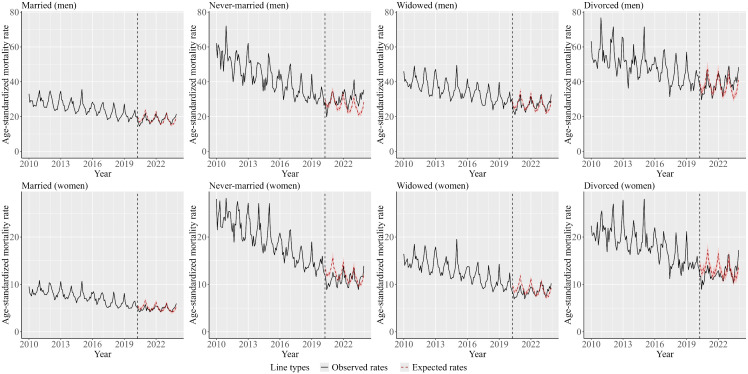
Monthly age-standardized mortality rates of respiratory diseases along with the expected values following the start of the pandemic by sex and marital status. The solid line shows the observed rate, while the vertical dotted line marks the start of the pandemic. The red dashed line represents the expected rates based on pre-pandemic data, with the shaded areas indicating the 95% CI.

Fig 4 presents the monthly age-standardized mortality rates for respiratory diseases and their expected values following the start of the pandemic, broken down by sex and marital status. The observed rates generally exceeded the expected rates for never-married men, while the opposite pattern was seen in never-married women.

**Fig 5:**
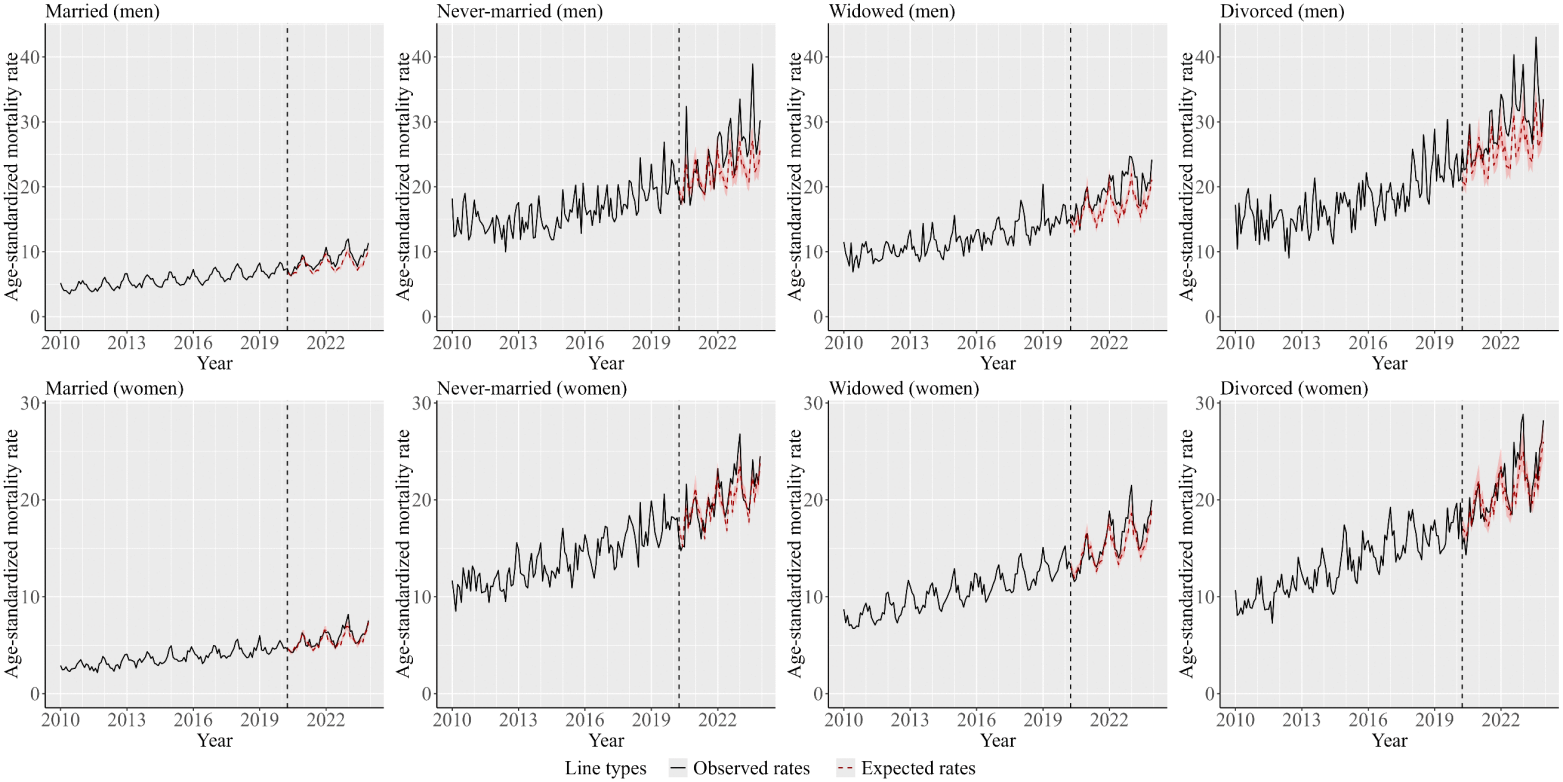
Monthly age-standardized mortality rates of ill-defined causes along with the expected values following the start of the pandemic by sex and marital status. The solid line shows the observed rate, while the vertical dotted line marks the start of the pandemic. The red dashed line represents the expected rates based on pre-pandemic data, with the shaded areas indicating the 95% CI.

Fig 5 illustrates the monthly age-standardized mortality rates for ill-defined causes and their expected values following the start of the pandemic, broken down by sex and marital status. A rising trend was evident both before and after the onset of the pandemic, regardless of marital status.

Table 1 displays the APC in mortality rates before and after the onset of the pandemic, categorized by sex, marital status, and causes of death. Overall, a declining trend was noted in all-cause mortality regardless of sex and marital status; however, an increasing trend emerged after the pandemic started. The APC for never-married persons was the lowest before the pandemic for both men and women, while that for married persons was the lowest after the pandemic began. For malignant neoplasms, a decreasing trend persisted even after the pandemic’s start across all groups. In terms of cardiovascular diseases, a significant increase was seen after the pandemic among never-married, widowed, and divorced men, but not among married men. Respiratory diseases showed trends similar to those of all causes, with an increase after the pandemic. For ill-defined causes, a significant upward trend was observed both before and after the pandemic across all sexes and marital statuses.

**Table 1:** APC in mortality rates before and after the onset of the pandemic, categorized by sex, marital status, and causes of death.

	Men		Women	
	Before the pandemic began (201001 − 202003)	After the pandemic began (202004 − 202312)	Before the pandemic began (201001 − 202003)	After the pandemic began (202004 − 202312)
Causes of death and marital status	APC (%) (95% CI)	APC (%) (95% CI)	APC (%) (95% CI)	APC (%) (95% CI)
All-cause				
Married	−2.5*** (−2.7, −2.4)	1.1** (0.4, 1.7)	−2.3*** (−2.5, −2.1)	1.1*** (0.4, 1.7)
Never-married	−3.1*** (−3.4, −2.8)	2.2*** (1.4, 3.0)	−2.8*** (−3.1, −2.5)	2.3*** (1.6, 3.1)
Widowed	−1.6*** (−1.8, −1.3)	3.3*** (2.4, 4.2)	−1.2*** (−1.4, −1.0)	3.5*** (2.6, 4.4)
Divorced	−2.4*** (−2.7, −2.2)	2.3*** (1.5, 3.1)	−1.5*** (−1.7, −1.3)	3.1*** (2.4, 3.8)
Malignant neoplasms				
Married	−2.4*** (−2.5, −2.3)	−2.2*** (−2.5, −1.9)	−1.4*** (−1.5, −1.3)	−1.0*** (−1.4, −0.5)
Never-married	−2.6*** (−2.9, −2.4)	−1.6*** (−2.3, −1.0)	−2.3*** (−2.5, −2.1)	−0.4 (−1.1, 0.4)
Widowed	−1.6*** (−1.8, −1.4)	−1.4*** (−1.8, −1.0)	−1.1*** (−1.2, −1.0)	−0.6*** (−1.0, −0.3)
Divorced	−2.1*** (−2.4, −1.8)	−1.8*** (−2.3, −1.3)	−1.2*** (−1.4, −1.0)	−0.4 (−1.3, 0.4)
Cardiovascular diseases				
Married	−3.8*** (−4.0, −3.6)	−0.4 (−1.1, 0.3)	−4.1*** (−4.3, −3.9)	−0.5 (−1.1, 0.2)
Never-married	−3.4*** (−3.8, −3.1)	1.4** (0.4, 2.4)	−4.6*** (−4.9, −4.3)	−0.9 (−2.1, 0.3)
Widowed	−3.1*** (−3.3, −2.9)	1.3** (0.4, 2.3)	−3.3*** (−3.4, −3.1)	0.1 (−0.6, 0.8)
Divorced	−3.2*** (−3.5, −2.9)	1.6*** (0.7, 2.6)	−3.0*** (−3.3, −2.8)	0.7 (−0.1, 1.5)
Respiratory diseases				
Married	−4.2*** (−4.5, −3.8)	1.6*** (0.8, 2.4)	−5.0*** (−5.5, −4.4)	1.5** (0.4, 2.7)
Never-married	−5.6*** (−6.0, −5.2)	2.6*** (1.4, 3.8)	−6.2*** (−6.7, −5.7)	1.8* (0.1, 3.6)
Widowed	−3.4*** (−3.7, −3.2)	2.4*** (1.7, 3.1)	−4.7*** (−5.1, −4.3)	2.8*** (1.9, 3.7)
Divorced	−3.7*** (−4.1, −3.3)	4.4*** (3.3, 5.6)	−4.5*** (−5.1, −3.9)	5.0*** (3.2, 6.8)
Ill-defined causes				
Married	5.1*** (4.8, 5.5)	8.5*** (7.6, 9.5)	5.6*** (5.2, 6.1)	8.7*** (7.4, 10.0)
Never-married	3.5*** (2.9, 4.1)	8.6*** (6.0, 11.3)	5.0*** (4.5, 5.4)	8.1*** (6.7, 9.5)
Widowed	5.6*** (5.4, 5.9)	9.6*** (8.5, 10.7)	5.5*** (5.3, 5.7)	8.5*** (7.5, 9.5)
Divorced	4.5*** (3.8, 5.2)	8.6*** (7.2, 9.9)	6.4*** (5.9, 7.0)	10.2*** (8.8, 11.7)

APC, annual percent change; CI, confidence intervals.

* p-value < 0.05, ** p-value < 0.01, *** p-value < 0.001.

Table 2 presents the P-score and the excess mortality rate following the start of the pandemic, broken down by sex, marital status, and causes of death. The age-standardized P-score for all-cause mortality significantly exceeded 0% across all groups, regardless of sex or marital status. Notably, the highest age-standardized P-scores for all-cause mortality were observed for never-married persons— 21.9% (95% CI: 19.1%, 24.7%) in men and 9.6% (95% CI: 6.9%, 12.3%) in women. Conversely, married men had the lowest value at 7.5% (95% CI: 6.5%, 8.6%), while married and divorced women had the lowest values, at 7.3% (95% CI: 5.9%, 8.8%) and 7.3% (95% CI: 5.9%, 8.9%), respectively. The confidence intervals for the age-standardized P-scores did not overlap among marital status groups in men, while those overlapped largely in women. The age-standardized P-score for malignant neoplasms was lower than that for all causes across both sexes and marital statuses, and never-married persons had the highest values, with the values of 8.7% (95% CI: 3.4%, 13.6%) for men and 3.0% (95% CI: 0.9%, 5.0%) for women. The highest age-standardized P-scores for cardiovascular diseases were observed in never-married persons, with the values of 26.3% (95% CI: 22.2%, 30.3%) for men and 11.2% (95% CI: 8.4%, 14.2%) for women. In addition, the confidence interval for never-married men did not overlap with those for the other groups. Regarding respiratory diseases, the highest age-standardized P-score was observed in never-married persons among men, at 14.6% (95% CI: 10.5%, 19.1%), and the confidence interval did not overlap with those for the other marital status groups. In contrast, the P-score for never-married persons was the lowest among women, at −6.9% (95% CI: −9.6%, −4.3%). For ill-defined causes, divorced persons had the highest age-standardized P-score at 16.5% (95% CI: 10.6%, 22.9%) among men. Among women, married and never-married persons showed the highest values, at 5.9% (95% CI: 2.8%, 9.2%) and 5.9% (95% CI: 2.1%, 9.6%), respectively. The age-standardized excess mortality rates per 100,000 person-years for divorced persons was the highest among men, with the value of 415.5 (95% CI: 388.3, 438.2), while that for never-married persons was the highest among women, with the value of 112.4 (95% CI: 82.2, 140.7).

**Table 2:** The P-score and the excess mortality rate following the start of the pandemic, broken down by sex, marital status, and causes of death.

Sex, causes of death, and marital status	Observed number of deaths	Expected number of deaths	P-score (95% CI)*	Age-standardized P-score (95% CI)*	Excess mortality rate per 100,000 person-years (95% CI)	Age-standardized excess mortality rate per 100,000 person-years (95% CI)
Men						
All-cause						
Married	1,692,036	1,575,221	7.4 (6.5, 8.4)	7.5 (6.5, 8.6)	102.0 (89.8, 114.0)	96.9 (84.3, 108.7)
Never-married	325,735	283,000	15.1 (13.0, 17.1)	21.9 (19.1, 24.7)	60.8 (53.4, 67.6)	393.7 (359.2, 426.6)
Widowed	569,563	517,874	10.0 (8.1, 11.9)	11.0 (9.1, 12.9)	765.1 (632.3, 894.5)	218.2 (169.0, 257.6)
Divorced	262,113	232,306	12.8 (10.6, 14.9)	15.0 (13.7, 16.4)	310.6 (262.4, 353.7)	415.5 (388.3, 438.2)
Malignant neoplasms						
Married	567,439	563,969	0.6 (−0.4, 1.5)	0.2 (−0.4, 0.9)	3.0 (−1.9, 7.5)	1.6 (−2.3, 5.1)
Never-married	71,499	69,601	2.7 (−0.1, 5.2)	8.7 (3.4, 13.6)	2.7 (−0.1, 5.0)	30.3 (11.9, 45.1)
Widowed	121,983	119,567	2.0 (0.6, 3.3)	1.0 (−0.8, 2.7)	35.8 (11.4, 57.8)	0.7 (−16.3, 12.6)
Divorced	71,443	70,133	1.9 (−0.6, 4.1)	3.8 (1.4, 6.3)	13.6 (−4.4, 29.5)	22.0 (4.7, 36.9)
Cardiovascular diseases						
Married	369,906	335,700	10.2 (8.6, 11.7)	10.0 (8.2, 11.8)	29.9 (25.5, 33.7)	28.2 (23.3, 32.7)
Never-married	86,190	73,111	17.9 (15.2, 20.4)	26.3 (22.2, 30.3)	18.6 (16.2, 20.8)	115.1 (103.0, 125.8)
Widowed	138,247	123,289	12.1 (9.4, 15.2)	12.9 (10.7, 15.3)	221.4 (176.5, 269.7)	58.0 (41.4, 69.5)
Divorced	68,064	59,001	15.4 (11.8, 18.6)	17.3 (14.2, 20.4)	94.5 (75.1, 111.5)	116.8 (99.1, 132.7)
Respiratory diseases						
Married	251,217	251,788	−0.2 (−2.7, 2.0)	−0.4 (−2.8, 1.9)	−0.5 (−6.0, 4.3)	−1.6 (−7.4, 3.6)
Never-married	32,568	30,130	8.1 (5.0, 10.6)	14.6 (10.5, 19.1)	3.5 (2.2, 4.5)	45.9 (34.8, 56.1)
Widowed	103,153	104,695	−1.5 (−4.4, 1.2)	1.5 (−2.2, 5.0)	−22.8 (−70.9, 18.2)	0.3 (−10.3, 7.2)
Divorced	28,879	27,749	4.1 (1.0, 6.9)	3.9 (0.3, 7.4)	11.8 (2.9, 19.4)	18.6 (3.8, 31.9)
Ill-defined causes						
Married	111,854	101,504	10.2 (8.0, 12.3)	10.6 (8.8, 12.4)	9.0 (7.2, 10.7)	9.9 (7.7, 11.9)
Never-married	31,906	29,378	8.6 (3.9, 12.8)	11.8 (6.1, 17.8)	3.6 (1.7, 5.1)	31.2 (17.0, 43.6)
Widowed	75,439	67,108	12.4 (10.0, 14.7)	14.9 (9.6, 19.8)	123.3 (101.5, 142.8)	28.9 (19.9, 34.5)
Divorced	24,231	21,381	13.3 (9.2, 16.6)	16.5 (10.6, 22.9)	29.7 (21.3, 36.0)	49.2 (33.4, 61.7)
Women						
All-cause						
Married	592,965	551,572	7.5 (6.3, 8.6)	7.3 (5.9, 8.8)	35.8 (30.3, 40.7)	45.1 (36.8, 52.9)
Never-married	182,593	165,132	10.6 (8.2, 12.8)	9.6 (6.9, 12.3)	32.4 (25.7, 38.5)	112.4 (82.2, 140.7)
Widowed	1,751,556	1,622,581	7.9 (6.0, 9.9)	9.0 (7.7, 10.2)	415.7 (321.5, 510.5)	89.1 (72.7, 100.9)
Divorced	200,592	186,636	7.5 (6.3, 8.7)	7.3 (5.9, 8.9)	92.8 (78.6, 106.8)	107.8 (87.3, 128.6)
Malignant neoplasms						
Married	219,766	216,663	1.4 (−0.3, 3.1)	1.5 (−0.0, 3.1)	2.7 (−0.5, 5.8)	2.6 (−0.5, 5.7)
Never-married	43,946	42,775	2.7 (0.8, 4.5)	3.0 (0.9, 5.0)	2.2 (0.6, 3.5)	11.4 (5.5, 16.6)
Widowed	279,769	274,301	2.0 (1.2, 2.9)	2.1 (1.4, 2.8)	17.6 (10.3, 25.1)	4.1 (−2.5, 8.8)
Divorced	56,880	56,238	1.1 (−0.9, 3.0)	1.5 (−0.8, 3.6)	4.3 (−3.6, 10.9)	3.4 (−4.7, 10.3)
Cardiovascular diseases						
Married	133,030	122,582	8.5 (6.6, 10.3)	8.1 (5.9, 10.2)	9.0 (7.1, 10.7)	11.7 (8.3, 14.7)
Never-married	40,899	37,037	10.4 (7.5, 13.2)	11.2 (8.4, 14.2)	7.2 (5.3, 8.9)	30.8 (22.8, 38.4)
Widowed	477,691	445,980	7.1 (4.8, 9.5)	7.6 (6.1, 9.1)	102.2 (70.3, 133.8)	17.2 (12.4, 20.6)
Divorced	48,683	45,421	7.2 (5.7, 8.4)	6.8 (5.4, 8.2)	21.7 (17.4, 25.2)	23.7 (17.4, 29.0)
Respiratory diseases						
Married	45,892	46,904	−2.2 (−7.5, 3.0)	−3.2 (−8.4, 2.3)	−0.9 (−3.2, 1.2)	−1.7 (−5.1, 1.3)
Never-married	15,962	17,214	−7.3 (−10.0, −5.0)	−6.9 (−9.6, −4.3)	−2.3 (−3.3, −1.6)	−11.0 (−14.9, −7.7)
Widowed	183,876	195,980	−6.2 (−9.8, −2.6)	−2.9 (−6.9, 0.7)	−39.0 (−64.5, −15.6)	−5.2 (−9.5, −2.1)
Divorced	17,197	18,048	−4.7 (−10.4, 1.0)	−5.3 (−10.7, 0.6)	−5.7 (−13.3, 1.2)	−8.2 (−18.6, 1.2)
Ill-defined causes						
Married	44,456	42,086	5.6 (2.9, 8.4)	5.9 (2.8, 9.2)	2.1 (1.1, 3.0)	3.1 (1.0, 4.9)
Never-married	28,910	28,119	2.8 (0.3, 5.1)	5.9 (2.1, 9.6)	1.5 (0.1, 2.6)	4.9 (−1.2, 10.0)
Widowed	390,138	369,418	5.6 (3.3, 8.1)	4.6 (1.4, 7.3)	66.8 (40.1, 93.9)	7.8 (1.9, 11.7)
Divorced	26,930	26,034	3.4 (−0.8, 7.3)	3.2 (−0.5, 6.6)	6.0 (−1.5, 12.2)	8.5 (−3.3, 18.6)

CI, confidence intervals.

* The P-score (%) indicates the percentage of excess number of deaths relative to the expected number of deaths.

Table 3 presents the P-score and the excess mortality rate for all-cause mortality following the start of the pandemic, broken down by sex, marital status, and age group. The results of the P-scores for persons aged 65 years or older were almost similar to those of persons of all ages. In persons aged <65 years, the age-standardized P-score for divorced persons was the highest among men, with the value of 18.8% (95% CI: 16.2%, 21.6%). In contrast, it was the highest in never-married persons among women, with the value of 19.4% (95% CI: 15.5%, 23.5%), and the confidence intervals did not overlap between married and never-married women.

**Table 3:** The P-score and the excess mortality rate for all-cause mortality following the start of the pandemic, broken down by sex, marital status, and age group.

Sex, age group, and marital status	Observed number of deaths	Expected number of deaths	P-score (95% CI)*	Age-standardized P-score (95% CI)*	Excess mortality rate per 100,000 person-years (95% CI)	Age-standardized excess mortality rate per 100,000 person-years (95% CI)
Men						
<65 years						
Married	113,963	99,892	14.1 (10.1, 18.4)	13.6 (10.1, 17.5)	20.3 (15.1, 25.5)	16.1 (12.0, 20.3)
Never-married	131,541	112,694	16.7 (14.7, 18.7)	17.8 (15.6, 20.0)	28.9 (25.8, 31.7)	52.8 (46.6, 58.3)
Widowed	4,355	3,637	19.7 (13.3, 25.3)	17.8 (9.3, 26.7)	110.9 (78.8, 135.6)	57.4 (12.8, 89.0)
Divorced	58,522	48,985	19.5 (16.4, 22.3)	18.8 (16.2, 21.6)	154.5 (133.9, 173.0)	105.8 (92.3, 117.7)
65 years or older						
Married	1,578,073	1,475,329	7.0 (6.0, 7.9)	7.0 (6.0, 8.0)	227.2 (196.3, 256.8)	265.4 (230.3, 299.6)
Never-married	194,194	170,306	14.0 (11.2, 16.8)	22.3 (19.3, 25.3)	468.1 (382.0, 546.9)	1104.3 (1000.5, 1208.7)
Widowed	565,208	514,237	9.9 (8.0, 11.8)	10.4 (8.9, 12.0)	834.4 (688.8, 976.7)	553.3 (479.9, 622.5)
Divorced	203,591	183,322	11.1 (8.6, 13.4)	14.6 (13.2, 16.1)	592.3 (473.0, 701.9)	1061.0 (978.5, 1130.9)
Women						
<65 years						
Married	79,233	71,049	11.5 (7.4, 15.3)	11.3 (7.2, 15.2)	10.8 (7.2, 13.8)	8.9 (5.7, 11.6)
Never-married	44,732	37,876	18.1 (14.4, 21.4)	19.4 (15.5, 23.5)	13.7 (11.2, 15.8)	27.6 (22.4, 32.4)
Widowed	6,494	5,421	19.8 (9.6, 28.9)	14.2 (6.4, 22.2)	53.4 (28.4, 72.4)	16.9 (−4.9, 32.3)
Divorced	26,800	23,882	12.2 (8.6, 15.5)	12.3 (9.0, 15.7)	30.2 (21.9, 37.2)	22.8 (17.0, 28.1)
65 years or older						
Married	513,732	480,523	6.9 (5.3, 8.3)	7.0 (5.2, 8.8)	83.7 (65.5, 99.2)	120.4 (91.7, 147.5)
Never-married	137,861	127,256	8.3 (5.5, 11.1)	8.7 (6.1, 11.5)	274.4 (185.8, 357.2)	289.2 (201.9, 370.2)
Widowed	1,745,062	1,617,160	7.9 (6.0, 9.9)	8.6 (7.3, 9.9)	440.8 (340.6, 542.4)	239.5 (198.3, 277.5)
Divorced	173,792	162,755	6.8 (5.3, 8.2)	6.9 (5.3, 8.5)	205.3 (163.7, 245.9)	284.8 (220.0, 347.8)

CI, confidence intervals.

* The P-score (%) indicates the percentage of excess number of deaths relative to the expected number of deaths.

S1 Table presents the P-score and the excess mortality rate following the start of the pandemic, broken down by sex, marital status, and causes of death using January 2020 as the time point at which the pandemic began. The results were similar to those of the analysis using April 2020 as the time point at which the pandemic began.

## Discussion

Research showed that excess mortality varied by marital status. Notably, never-married persons experienced the highest age-standardized P-score for all-cause mortality among both men and women, and the difference from the other marital status group was significant in men. Before the pandemic, the largest decrease in mortality rates was observed among never-married persons, while after the pandemic started, their increase in mortality rate was not the highest. This suggests that the significant decline in mortality among never-married persons before the pandemic contributed to their highest age-standardized P-score during the pandemic.

A previous study showed that the mortality risk for COVID-19 was highest among never-married persons in Japan [19], contributing to the highest level of excess all-cause mortality among this group. A study in South Korea also found that excess mortality (%) was highest among single (never-married) persons across marital statuses [9]. It was noted that the effects of restricted healthcare access during the pandemic on the continuity of care for chronic diseases could be more pronounced among persons with lower socioeconomic status [9]. A study from the United States found that health declines during the pandemic were most significant among never-married persons, with health deterioration milder among those previously married than among never-married persons [30]. It was noted that previously married persons had longer marriage durations and greater resources than never-married persons [30]. Additionally, research in Japan indicated that single older adults living alone tended to experience unhealthy lifestyle changes during the pandemic more compared to married older adults [16]. Another study in the UK also reported that older single men living alone experienced worsening physical and mental health during this period [17]. Our study showed that an increase in all-cause mortality rate was the lowest among married persons after the starts of the pandemic, which aligned with the findings of those previous studies. In contrast, the highest age-standardized P-score for all-cause mortality was observed in divorced persons among men aged <65 years, although the difference from the other marital status groups was not evident. It might be caused by the difference in the relationship between socioeconomic status and marital status depending on ages. The proportion of never-married persons decreases with age, and being never-married in older ages was shown to be particularly associated with lower socioeconomic status among men [31]. Therefore, it is possible that never-married men were particularly socioeconomically disadvantaged among men aged 65 years or older. In contrast, never-married women experienced the highest age-standardized P-score for all-cause mortality among women aged <65 years, and there was a significant difference in the values between never-married women and married women. A previous study showed that the degree of excess suicides was particularly large among young women in 2020–2021 in Japan [32]. In addition, another study in Japan showed that the impact of the pandemic on household income and psychological distress was particularly large among socially vulnerable persons [33]. Moreover, a study in Germany showed that psychological responses to the pandemic differed depending on sociodemographic groups, and some characteristics, including women, younger persons, and lower income groups, were associated with mental deterioration during the pandemic [34]. Therefore, it is possible that among women aged <65 years, never-married women particularly experienced social and psychological hardships during the pandemic.

Regarding cause-specific mortality, the age-standardized P-score for cardiovascular diseases was particularly large among never-married persons in both men and women. Several studies in other countries have linked socioeconomic status with cardiovascular risk during the pandemic [35–37]. For example, a US. study found that intracerebral hemorrhage mortality rates increased, especially among lower-income groups [37]. Being never-married is also a risk factor for conditions like diabetes and hypertension [38], with hypertension being more common among unmarried persons in Japan compared to married ones [15]. Furthermore, lower socioeconomic status is associated with higher rates of hypercholesterolemia and diabetes in Japan [39,40]. These factors suggest that a greater prevalence of pre-existing health conditions may partly explain the higher excess mortality among never-married persons. Additionally, the age-standardized P-score for respiratory diseases for never-married persons was highest among men and lowest among women. The reduction in mortality and hospitalization rates for respiratory diseases during the pandemic has been noted in Japan and other countries [41–43]. It was pointed out that some preventive measures during the pandemic might have contributed to the reduction of mortality due to respiratory diseases [43]. Multiple studies indicated that women tended to take preventive, health-seeking, or infection-control behaviors, such as wearing a mask and self-restraint from social behaviors, compared with men during the pandemic [44–47]. A study in Japan indicated that women and married persons were associated with self-restraint from social behaviors during the pandemic [44], while it was not investigated whether the relationship between self-restraint and marital status differed by sex. In addition, the proportion of Chronic Obstructive Pulmonary Disease among causes of death for respiratory diseases is small in women than in men in Japan [48], and the difference in causes of death by sex might have affected the excess mortality. It is important to explore the reasons for these differences in future research studies, particularly with respect to sociodemographic factors. For ill-defined causes, the difference in the percentage of excess mortality among marital status groups was not evident in men as well as in women, and an increase in the mortality rate during the pandemic was observed in all the marital status groups. Senility accounts for a large part of the ill-defined causes in Japan [48], and the number of senility deaths in nursing home or long-term care facilities increased over the decades in Japan [49]. An increasing number of patients with incurable diseases moved out to facilities from hospitals before dying was hypothesized as a reason in the previous study [49].

A previous study in Japan showed that the decline in the age-standardized mortality rate from 2000 to 2015 for never-married persons was greatest among persons aged 40 years or older [20]. However, our study showed that the trend changed after the pandemic began. It is important to monitor whether the mortality trend returns to the pre-pandemic pattern in future studies. In addition, it is meaningful to investigate changes in other health indicators, including disease prevalence and incidence and hospitalization rates, to understand differences in the pandemic’s impact by marital status. Moreover, marital status is a socioeconomic factor which is related to other factors such as living arrangement and income, and whether marital status is a causal factor of excess mortality or not is not certain. For example, the degree of excess mortality may have differed between never-married persons living alone and those living with others. We could only use marital status as the social characteristics in this study, and the other factors may have had a larger role in the excess mortality. Therefore, conducting a study taking into account other social characteristics is important in the future.

Our study has some limitations. First, we relied on population data from the Census and used estimates for years when the Census was not conducted, while the trend in the population by marital status may have changed during the pandemic. If the never-married population increased than expected due to the decrease in marriage rate by the pandemic [50], that can cause the overestimation of the mortality rate for those population. The marriage rate in older ages is low in Japan [48], and it is considered that the pandemic did not have a large effect on the population of never-married persons in older ages. However, it is possible that the excess mortality for never-married persons during the pandemic was actually smaller than that shown in this study, particularly in persons aged <65 years. Additionally, the population data for married persons included de facto married persons, whereas the mortality data for married persons accounted only for legally married persons. This discrepancy may lead to an underestimation of the mortality rate among married persons. However, the proportion of the de facto married persons among the sum of legally and the de facto married persons is relatively small in Japan. According to a survey conducted by the Cabinet Office in 2021, the proportion of the de facto married persons among all the married persons in their sixties was 1.7% in men and 4.0% in women [51]. In addition, whether including de facto married persons as married persons in the Census affected the results of excess mortality or not is not certain because there is no evidence indicating the change in the proportion of those persons after the pandemic. Furthermore, we lacked detailed information for each mortality record, such as living arrangement, income, and comorbidity, so we could not analyze changes in marital status characteristics over time. Conversely, we used comprehensive Vital Statistics data for all of Japan, allowing our findings to reflect overall mortality trends by marital status.

## Conclusions

The increase in mortality rates for married persons was the smallest after the pandemic started, in both men and women. In addition, the age-standardized P-score for all-cause mortality in never-married persons was the highest among both genders, while it was lowest for married persons. Particularly, the age-standardized P-score for all-cause mortality for never-married persons was significantly higher than those of the other marital status groups in men, while the difference among marital status groups was smaller in women. Therefore, the largest percentage of excess mortality was observed among never-married persons during the pandemic, particularly in men, highlighting the need to monitor this trend.

## Supporting information

S1 Table P-score and the excess mortality rate following the start of the pandemic, broken down by sex, marital status, and causes of death using January 2020 as the time point at which the pandemic began. (PDF)

S2 Table Dataset used in the analysis. (CSV)
