# Supplementary material for: Difference in excess mortality during the COVID-19 pandemic depending on marital status in Japan
Source: PLoS One. 2026 Jul 17;21(7):e0354263. doi: 10.1371/journal.pone.0354263 (PMC13379106; doi:10.1371/journal.pone.0354263)
Supplement: S1 Table — (PDF) [file pone.0354263.s001.pdf]

S1 Table. P−score and the excess mortality rate following the start of the pandemic, broken down by sex, marital status, and causes of death using January 2020 as the time point at which the pandemic began

Sex, causes of death, and marital status	Observed number of deaths	Expected number of deaths	P-score (95% CI)*	Age-standardized P-score (95% CI)*	Excess mortality rate per 100,000 person-years (95% CI)	Age-standardized excess mortality rate per 100,000 person-years (95% CI)
Men						
All-cause						
Married	1,805,265	1,697,627	6.3 (5.4, 7.2)	6.3 (5.4, 7.3)	88.1 (76.0, 99.3)	82.6 (70.2, 94.1)
Never-married	346,403	303,574	14.1 (11.7, 16.3)	20.6 (17.7, 23.8)	57.1 (48.6, 64.9)	372.3 (330.4, 410.7)
Widowed	606,658	559,379	8.5 (6.3, 10.4)	9.6 (7.9, 11.2)	656.2 (498.9, 792.1)	190.1 (148.6, 223.8)
Divorced	278,805	248,851	12.0 (9.7, 14.2)	13.5 (12.0, 15.1)	293.0 (241.0, 338.5)	384.0 (341.7, 420.6)
Malignant neoplasms						
Married	605,323	601,543	0.6 (−0.4, 1.6)	0.2 (−0.5, 1.0)	3.1 (−1.8, 7.9)	1.6 (−2.3, 5.4)
Never-married	75,972	73,738	3.0 (−0.0, 6.1)	9.6 (4.5, 14.8)	3.0 (−0.0, 5.8)	32.8 (16.3, 47.6)
Widowed	129,914	127,496	1.9 (0.6, 3.3)	1.0 (−0.5, 2.4)	33.6 (10.7, 57.4)	0.4 (−16.7, 12.9)
Divorced	76,036	74,266	2.4 (−0.1, 4.6)	3.8 (0.8, 6.5)	17.3 (−1.0, 33.0)	23.3 (4.2, 40.0)
Cardiovascular diseases						
Married	396,633	364,677	8.8 (7.3, 10.2)	8.6 (6.9, 10.3)	26.2 (22.1, 30.1)	24.6 (20.1, 29.0)
Never-married	92,006	79,197	16.2 (11.9, 20.3)	23.5 (18.4, 28.9)	17.1 (13.1, 20.8)	105.6 (85.9, 123.6)
Widowed	148,079	134,068	10.5 (7.2, 14.1)	11.3 (8.2, 14.3)	194.5 (138.4, 253.6)	51.3 (35.4, 61.7)

Divorced	72,847	63,581	14.6 (10.6, 18.6)	15.6 (11.6, 19.4)	90.6 (68.5, 111.6)	109.3 (85.4, 130.3)
Respiratory diseases						
Married	269,861	273,883	−1.5 (−4.2, 1.4)	−1.6 (−4.1, 1.0)	−3.3 (−9.6, 3.0)	−4.7 (−11.3, 1.6)
Never-married	34,830	32,725	6.4 (3.8, 8.8)	11.6 (8.4, 14.7)	2.8 (1.7, 3.8)	37.1 (28.5, 44.6)
Widowed	110,632	114,201	−3.1 (−5.5, −1.0)	0.8 (−3.0, 5.1)	−49.5 (−88.6, −15.3)	−4.1 (−13.4, 2.4)
Divorced	30,872	30,098	2.6 (−1.1, 5.9)	2.1 (−1.6, 6.1)	7.6 (−3.3, 16.8)	10.4 (−6.3, 25.7)
Ill-defined causes						
Married	117,890	109,450	7.7 (5.9, 9.5)	8.0 (6.2, 9.9)	6.9 (5.4, 8.4)	7.6 (5.8, 9.4)
Never-married	33,581	31,207	7.6 (1.8, 12.4)	11.7 (5.7, 17.8)	3.2 (0.8, 4.9)	30.5 (15.6, 42.9)
Widowed	79,520	71,980	10.5 (8.4, 12.4)	12.0 (8.8, 14.4)	104.6 (85.5, 121.9)	23.5 (17.5, 26.5)
Divorced	25,412	22,879	11.1 (6.2, 14.8)	12.9 (6.7, 19.5)	24.8 (14.5, 32.0)	39.5 (21.1, 53.2)
Women						
All-cause						
Married	631,582	595,144	6.1 (4.9, 7.3)	5.7 (4.7, 6.8)	29.6 (24.1, 35.0)	35.8 (29.4, 41.9)
Never-married	194,248	178,068	9.1 (7.3, 11.0)	8.0 (5.8, 10.3)	28.1 (22.9, 33.4)	94.9 (69.6, 120.4)
Widowed	1,864,977	1,751,882	6.5 (4.8, 8.0)	7.7 (6.0, 9.3)	341.7 (256.9, 416.6)	76.6 (51.3, 96.0)
Divorced	213,259	200,219	6.5 (4.7, 8.2)	6.2 (4.6, 7.9)	81.4 (60.2, 100.4)	91.9 (68.3, 114.8)
Malignant neoplasms						
Married	234,184	230,627	1.5 (0.2, 2.8)	1.6 (0.3, 3.0)	2.9 (0.3, 5.2)	2.8 (0.5, 4.8)
Never-married	46,666	45,692	2.1 (0.3, 3.8)	2.9 (0.9, 4.7)	1.7 (0.3, 3.0)	9.7 (3.6, 15.1)
Widowed	297,979	291,698	2.2 (1.1, 3.2)	2.5 (1.3, 3.5)	19.0 (9.7, 27.9)	6.4 (−0.1, 10.9)
Divorced	60,572	59,340	2.1 (−0.1, 3.9)	2.4 (1.1, 3.8)	7.7 (−0.5, 14.1)	6.9 (1.1, 11.6)

Cardiovascular diseases						
Married	142,390	133,853	6.4 (5.2, 7.4)	5.9 (4.7, 7.1)	6.9 (5.7, 8.0)	8.5 (6.4, 10.4)
Never-married	43,787	40,359	8.5 (5.9, 10.9)	9.3 (6.9, 11.7)	6.0 (4.2, 7.5)	25.8 (19.0, 31.8)
Widowed	511,473	484,586	5.5 (3.1, 7.9)	6.3 (4.2, 8.2)	81.2 (46.3, 113.8)	14.2 (5.8, 20.6)
Divorced	52,050	49,132	5.9 (4.3, 7.2)	5.5 (4.0, 6.9)	18.2 (13.5, 21.8)	19.2 (13.4, 24.0)
Respiratory diseases						
Married	49,336	51,245	−3.7 (−7.5, −0.1)	−4.7 (−8.3, −1.2)	−1.5 (−3.2, −0.0)	−2.9 (−5.4, −0.7)
Never-married	17,251	18,788	−8.2 (−12.4, −4.2)	−7.8 (−12.2, −3.1)	−2.7 (−4.2, −1.3)	−13.0 (−19.4, −7.3)
Widowed	198,005	214,772	−7.8 (−11.4, −4.0)	−3.9 (−7.7, −0.1)	−50.7 (−76.6, −24.8)	−6.8 (−11.1, −3.1)
Divorced	18,441	19,760	−6.7 (−11.0, −2.7)	−7.2 (−11.4, −2.9)	−8.2 (−14.2, −3.2)	−11.9 (−20.2, −4.4)
Ill-defined causes						
Married	46,815	45,415	3.1 (1.0, 4.9)	3.4 (1.4, 5.3)	1.1 (0.4, 1.8)	1.6 (−0.1, 3.1)
Never-married	30,549	30,007	1.8 (−0.3, 3.5)	5.0 (1.0, 9.3)	0.9 (−0.2, 1.8)	2.5 (−2.2, 6.2)
Widowed	411,970	397,131	3.7 (1.5, 6.1)	3.2 (0.1, 5.9)	44.8 (18.0, 71.4)	4.8 (−1.4, 8.4)
Divorced	28,374	27,793	2.1 (−2.5, 6.1)	1.8 (−1.8, 5.2)	3.6 (−4.6, 10.1)	5.3 (−7.6, 15.7)

CI, confidence intervals

* The P-score (%) indicates the percentage of excess number of deaths relative to the expected number of deaths.
